# Comparison of the Effect of Pump Flow Type (Pulsatile or Non-Pulsatile) on Postoperative Neurocognitive Functions in Coronary Artery Surgery

**DOI:** 10.21470/1678-9741-2023-0345

**Published:** 2024-09-03

**Authors:** Ferhat Borulu, Bilgehan Erkut

**Affiliations:** 1 Department of Cardiovascular Surgery, Faculty of Medicine, Ordu University, Ordu, Turkey; 2 Department of Cardiovascular Surgery, Faculty of Medicine, Atatürk University, Erzurum, Turkey

**Keywords:** Cardiopulonary Bypass, Patient Discharge, Pulsative Flow, Coronary Artery Bypass, Cognition, Mental Status and Dementia Test, Intelligence Tests, Critical Care

## Abstract

**Introduction:**

The effect of pump flow type on perfusion in coronary surgery using cardiopulmonary bypass (CPB) is discussed. We aimed to evaluate the effect of pump flow type on cognitive functions with neurocognitive function tests.

**Methods:**

One hundred patients who underwent isolated coronary artery bypass surgery between November 2020 and July 2021 were divided into two equa groups. Groups were formed according to pump flow type pulsatile (Group 1) and non-pulsatile (Group 2). Clock drawing test (CDT) and standardized mini mental test (SMMT) were performed on the patients in both groups in the preoperative period, on the 1st preoperative day, and on the day before discharge. Neurocognitive effects were compared with all follow-up parameters.

**Results:**

There was no difference between the groups in terms of demographic data and in terms of neurocognitive tests performed before the operation. SMMT on postoperative day 1 (Group I: 27.64 ± 1.05; Group II: 24.44 ± 1.64; P=0.001) and CDT (Group I: 5.4 ± 0.54; Group II: 4 .66 ± 0.52; P=0.001), and SMMT on the day before discharge (Group I: 27.92 ± 1.16; Group II: 24.66 ± 1.22; *P*=0.001) and CDT (Group I: 5 It was calculated as .66 ± 0.48; Group II: 5.44 ± 0.5; *P*=0.001). The duration of intensive care and hospitalization were higher in the non-pulsatile group.

**Conclusion:**

We think that the type of pump flow used in coronary artery bypass surgery using CPB is effective in terms of neurocognitive functions and that pulsatile flow makes positive contributions to this issue.

## INTRODUCTION

Open heart surgery technique brings with it the risks of postoperative complications due to cardiopulmonary bypass (CPB) pump and extracorporeal circulation (ECC). Although various methods have been developed to prevent the undesirable effects of CPB, the frequency of cerebrovascular complications is still higher. The presence of these complications continues to be important, especially since patients in the advanced age groups are more likely to be operated on.

Central nervous system complications after CPB are one of the most serious complications. Although complications such as neuropsychological disorders, cognitive dysfunction, and delirium are common after operations using ECC, ischemic stroke constitutes the most severe clinical picture. And although there has been a decrease in stroke and death rates due to developments in anesthesia, CPB techniques, and surgical techniques in recent years, cognitive dysfunction can be observed in nearly half of the patients^[1]^. These changes, especially postoperative delirium, prolong the length of stay in the intensive care unit and in hospital^0^. Although the mechanism in the formation of neurological complications has not been fully clarified, it is thought to be multifactorial^0^. Three important factors stand out: hypoperfusion, microembolism and systemic inflammatory response^[4]^. Whatever the reason may be, the detection of these undesirable situations cannot always be detected by classical radiological methods. There are studies on the causes of these problems, which are tried to be detected by neurocognitive tests.

In this study, we aimed to investigate whether the operation of the heart-lung pump with pulsatile or non-pulsatile flow has an effect on the development of these complications after CPB.

## METHODS

The approval of Atatürk University Faculty of Medicine Clinical Research Ethics Committee (dated 01.10.2020, decision no. 19) was obtained. One hundred patients between the ages of 25 and 65 years who were going to undergo isolated coronary artery bypass surgery in the Faculty of Medicine Research Hospital, Department of Cardiovascular Surgery, were informed about the study and included in the study after obtaining consent forms. Patients who had a previous cerebrovascular accident or were using drugs that could affect cerebral functions were not included in the study. In addition, patients with > 50% stenosis in the carotid arteries and without indication for surgery were excluded from the study. Patients who developed a complication that was too serious to perform the neurocognitive tests, which was the main element of the study, after the operation were excluded from the study.

### Study Methods

Standard clock drawing test (CDT) and standardized mini mental test (SMMT) were applied to evaluate neurocognitive functions one day before the operation day for patients who applied to our clinic and were planned for isolated coronary bypass surgery and met the working conditions. Patients in whom the pump was operated in pulsatile form were included in Group 1, and patients in which the pump was operated in non-pulsatile form were included in Group 2. Apart from these tests, no other procedure or examination was performed in terms of routine preoperative preparations of the patients. The same drugs were used for induction of anesthesia in all patients. At entry to CPB, before induction of anesthesia. Vital signs were recorded at the 5^th^ minute of CPB, when the cross-clamp was placed, at the 10^th^ and 20^th^ minutes of the cross-clamp, when the cross-clamp was removed, at the end of CPB, and at the end of the operation. At the same time, blood gases were taken from the patients and metabolites, pH, oxygenation, and lactate levels were recorded. The routine laboratory data of the patients before the operation, on the 1^st^ and on the 5^th^ postoperative days, were also recorded. In CPB, the total pump flow was adjusted to be 2,4 lt/m^2^/min.

It was tried to keep the hematocrit in the pump as 24-26%. Mean blood pressure value in the range of60-70 mmHg was achieved with pump flow and medical interventions. Alpha stat pH monitoring regimen was applied to all patients and nasopharyngeal body temperature was reduced to a minimum of 32 °C. The patients were divided into two groups with equal numbers. In one of the groups, the pump was operated in pulsatile mode and in the other group in non-pulsatile form. A roller pump was used as the pump. The current was converted to pulsatile form with the digital button on the device. Electrocardiogram frequency and current ratio were adjusted independently of each other. In patients using pulsatile flow, the heart rate was set at 60 rpm, the pulse width was 40-50%, and the basal flow amount was 40% while on the pump.

Postoperative intensive care and service follow-ups of the patients were performed as standard. Operational data such as cross-clamping time, CPB time, extubation time, intensive care unit length, hospitalization time, blood transfusion amount, drainage amount, and postoperative complications were recorded.

Tests for the analysis of neurocognitive functions, which formed the basis of the study, were performed again on the postoperative 1st day and the day before discharge and recorded.

### The Clock Drawing Test

This test is performed on the basis of patients drawing a desired clock. They are asked to place the numbers in the appropriate places, to mark the said time correctly on the paper. It is essential to control structural praxis, comprehension, and planning ability in patients. Scoring is done out of 6. Scores < 4 indicate cognitive dysfunction. Scoring is done as follows:

- If the position of number 12 is correct: 3 points
- If all 12 numbers are noted: 1 point
- If the hour and minute hands are drawn: 1 point
- If the time requested from the patient is marked correctly: 1 point

Its advantages are that it is a short test, requires less time to be administered, and has a high negative predictive value. The disadvantage is that the test scoring is subjective. The results have a high level of false negativity as a disadvantage^[5,6]^.

### Standardized Mini Mental Test

The SMMT is a test used to measure cognitive performance. It has limited specificity to distinguish clinical syndromes. However, it can be used for a general assessment of cognition as a short, useful, and standardized method. It consists of five main themes: orientation, recording memory, attention and calculation, recall, and language. It consists of eleven items. The total score on the test is 30.

### Statistics

In our study, we used the NCSS (Number Cruncher Statistical System) 2007 (Kaysville, Utah, United States of America) program to analyze the data. While evaluating the data, we evaluated the descriptive statistical methods (mean, standard deviation, median, frequency, ratio, minimum, maximum) and the distribution of the data with the Shapiro-Wilk test. We used the Mann-Whitney U test to compare two sets of quantitative data. Chi-square analysis was used to understand the relationship between qualitative data. Statistical significance was set at *P*<0.01 and *P*<0.05 levels.

## RESULTS

The men in the pulsatile group were 50.7% and 49.3% in the nonpulsatile group. According to the New European System for Cardiac Operative Risk Evaluation (or EuroSCORE) II calculation, it was evaluated as 1.33 ± 0.49 in the pulsatile group and 1.25 ± 0.44 in the non-pulsatile group. There was no statistical difference between the groups (*P*=0.415). The mean age of the patients was 60.02 ± 9.16 in the pulsatile group and 61.72 ± 8.76 in the non-pulsatile group. Body mass index was 27.57 ± 2.9 in the pulsatile group and 27.8 ± 3.1 in the non-pulsatile group. There was no difference between the groups in characteristics such as hypertension, diabetes mellitus, hyperlipidemia, chronic obstructive pulmonary disease, and smoking (Table 1). The preoperative ejection fractions of the patients were 51.62 ± 5.97 in the pulsatile group, while it was 49.96 ± 5.89 in the non-pulsatile group, no difference was observed.

**Table 1:** Demographic data of the patients.

Parameters	Group 1 (pulsatile) (n: 50)	Group 2 (non-pulsatile) (n: 50)	*P*-value
Age (years)	60.02 ± 9.16	61.72 ± 8.76	0.289*
Male sex (n/%)	37 (74%)	36 (72%)	0.500**
Female sex (n/%)	13 (26%)	14 (28%)	0.500**
BMI (kg/m^2^)	27.57 ± 2,9	27.8 ± 3.01	0.833*
BSA (m^2^)	1.95 ± 0.17	1.97 ± 0.16	0.588*
Hypertension (n/%)	17 (34%)	20 (40%)	0.339**
DM (n/%)	20 (40%)	16 (32%)	0.266**
Cigarette (n/%)	20 (40%)	17 (34%)	0.339**
Hyperlipidemia (n/%)	17 (34%)	12 (24%)	0.189**
Preoperative EF (%)	51.62 ± 5.97	49.96 ± 5.89	0.170*
CCPD (n/%)	4 (8%)	8 (16%)	0.178**
PAD (n/%)	2 (4%)	4 (8%)	0.339**
CRF (n/%)	1 (2%)	2 (4%)	0.554**
EuroSCORE II	1.33 ± 0.49	1.25 ± 0.44	0.415*

BMI=body mass index; BSA=body surface area; CCPD=chronic obstructive pulmonary disease; CRF=chronic renal failure; DM=diabetes mellitus; EF=ejection fraction; EuroSCCRE=European System for Cardiac Operative Risk Evaluation; PAD=peripheral artery disease

* Mann-Whitney U test; **Chi-square analysis

When the operative data were examined, the mean perfusion flow was 4101 ± 391.62 ml/kg-min, the mean perfusion time was 85.4 ± 18.47 minutes, and the mean aortic cross-clamping time was 40.18 ± 10.28 minutes in the pulsatile group; the mean perfusion flow was 4085 ± 297.63 ml/kg min, perfusion time was 91.74 ± 17.17 minutes, and aortic cross-clamping time was 47.1 ± 14.67 minutes in the non-pulsatile group. Perfusion time and perfusion flow data were similar between the groups (*P*=0.305 and *P*=0.324, respectively), but aortic cross-clamping times were slightly longer in the non-pulsatile group. The maximal cooling temperature was made up to similar degrees in both groups (Table 2). The measured intraoperative body temperatures were recorded as 32.52 ± 0.9 in the pulsatile group and 31.66 ± 0.85 in the non-pulsatile group, it was similar in the two groups.

**Table 2:** Intraoperative data.

Parameters	Group 1 (pulsatile) (n: 50)	Group 2 (non-pulsatile) (n: 50)	*P*-value
CPB time (min.)	91.41 ± 17.47	93.74 ± 18.17	0.305*
Cross-clamping time (min.)	44.18 ± 10.28	47.1 ± 14.67	0.411*
Number of vessels (n)	3.06 ± 0.82	3.15 ± 0.89	0.301
Body temperature (°C)	32.52 ± 0.9	31.66 ± 0.85	0.864
Perfusion current (ml/min)	4130 ± 292.75	4095 ± 301.45	0.324
Pre-induction MAP (mmHg)	67.8 ± 10.19	68.56 ± 11.66	0.928
CPB entry MAP (mmHg)	67.18 ± 7.1	68.64 ± 10.53	0.661
5^th^ minute CPB MAP (mmHg)	62.24 ± 6.77	60.9 ± 8.25	0.318
10^th^ minute cross-clamping MAP (mmHg)	61.28 ± 7.74	59.22 ± 9.53	0.125
20^th^ minute cross-clamping MAP (mmHg)	60.44 ± 8.5	58.68 ± 9.3	0.427
CPB exit MAP (mmHg)	59.46 ± 8.63	59.74 ± 8.16	0.839

CPB=cardiopulmonary bypass; MAP=mean arterial pressure

* Mann-Whitney U test

Generally, no significant difference was observed in the blood gas data of the patients before induction, at CPB entry, aortic cross-clamping at the 5^th^ minute, aortic cross-clamping at the 10^th^ minute, aortic cross-clamping at the 20^th^ minute, and CPB exit. Small differences were seen in some blood gas parameters. Pre-induction sodium value was lower in the pulsatile group (*P*=0.02). At the 10^th^ minute of aortic cross-clamping, CO_2_ and saturation were higher in group 1 (*P*=0.004 and *P*=0.032, respectively). At the 20^th^ minute of aortic cross-clamping, CO_2_ was again higher in group 1 (*P*=0.007). After CPB, only blood sugar levels were measured to be higher in the pulsatile group (*P*=0.004) (Table 3).

**Table 3:** Intraoperative blood gas data.

Parameters	Group 1 (pulsatile) (n: 50)	Group 2 (non-pulsatile) (n: 50)	*P*-value
**CPB entry**			
PH	7.32 ± 0.06	7.32 ± 0.05	0.754
pO_2_	244.84 ± 53.44	240.18 ± 57.09	0.484
pCO_2_	43.09 ± 5.09	43.62 ± 5.73	0.756
Saturation	99.06 ± 0.64	99.01 ± 0.66	0.361
Na	137.48 ± 2.63	137.2 ± 3.06	0.626
Glucose	158.74 ± 37.47	147.61 ± 41.9	0.093
Lactate	1.95 ± 0.72	1.72 ± 0.72	0.077
Hgb	6.91 ± 1.14	7.12 ± 1.43	0.473
Hct	21.66 ± 3.76	22.22 ± 4.34	0.544
**Cross-clamping (5 min.)**			
PH	7.33 ± 0.04	7.34 ± 0.06	0.396
pO_2_	186.08 ± 44.67	187.36 ± 45.19	0.953
pCO_2_	39.62 ± 5.99	39.29 ± 7.94	0.410
Saturation	98.68 ± 0.79	98.66 ± 1.55	0.262
Na	138.56 ± 2.87	138.48 ± 3.54	0.523
Glucose	205.96 ± 38.39	195.28 ± 43.96	0.288
Lactate	2.76 ± 0.97	3.05 ± 2.46	0.972
Hgb	7.82 ± 1.08	7.97 ± 1.34	0.831
Hct	23.95 ± 2.85	24.68 ± 4.11	0.565
**Cross-clamping (10 min.)**			
pH	7.3 ± 0.07	7.32 ± 0.08	0.065
pO_2_	208.1 ± 50.35	214.58 ± 53.99	0.345
pCO_2_	43.78 ± 5.48	41.22 ± 5.32	0.032*
Saturation	99.16 ± 1.09	98.5 ± 1.62	0.004**
Na	140.24 ± 2.85	140.68 ± 4.07	0.751
Glucose	178.28 ± 32.73	181.38 ± 36.26	0.730
Lactate	2.63 ± 0.79	2.85 ± 1	0.292
Hgb	6.62 ± 0.49	6.61 ± 0.63	0.779
Hct	21.1 ± 1.35	20.99 ± 1.72	0.828
**Cross-clamping (20 min.)**			
pH	7.28 ± 0.06	7.31 ± 0.07	0.005**
pO_2_	203.76 ± 44.83	194.98 ± 42.7	0.244
pCO_2_	42.99 ± 5.52	40.11 ± 4.79	0.007**
Saturation	99.22 ± 1.08	99.12 ± 1.05	0.112
Na	139.12 ± 2.02	139.04 ± 3.79	0.540
Glucose	181.62 ± 44.2	182.1 ± 29.89	0.631
Lactate	3.07 ± 0.92	2.91 ± 0.99	0.415
Hgb	6.84 ± 0.48	6.94 ± 0.6	0.258
Hct	21.46 ± 1.31	21.53 ± 3.18	0.227
**CPB exit**			
pH	7.34 ± 0.05	7.36 ± 0.06	0.138
pO_2_	192,76 ± 29,94	188.56 ± 32.26	0.329
pCO_2_	39.85 ± 5.13	38.66 ± 5.54	0.334
Saturation	99.16 ± 0.57	98.92 ± 0.78	0.110
Na	138.82 ± 1.72	139.2 ± 1.98	0.311
Glucose	208.88 ± 39.38	185.32 ± 42.34	0.004**
Lactate	2.86 ± 1.13	2.85 ± 1.42	0.588
Hgb	7.85 ± 0.83	8.01 ± 0.96	0.541
Hct	24.11 ± 2.44	24.99 ± 2.79	0.164

CPB=cardiopulmonary bypass; Hct=hematocrit; Hgb=hemoglobin

Mann-Whitney U Test

* *P*<0.05; **P<0.01

Considering the tests for measuring neurocognitive functions, which is the main starting point of the study, there was no difference between the groups in the preoperative CDT and SMMT measurements. The measurement values of CDT on the first postoperative day were 5.4 ± 0.54 in the pulsatile group and 4.66 ± 0.52 in the non-pulsatile group (*P*=0.001). SMMT values on the first postoperative day were 27.64 ± 1 in the pulsatile group. In the nonpulsatile group, it was 24.44 ± 1.64 (*P*=0.001). In the pre-discharge measurements of the same tests, CDT was 5.66 ± 0.48 in the pulsatile group and 5.44 ± 0.5 in the non-pulsatile group (*P*=0.001). SMMT was measured as 27.92 ± 1.16 in the pulsatile group and 24.66 ± 1.12 in the non-pulsatile group (*P*=0.312) (Table 4).

**Table 4:** Neurocognitive test results between groups.

		Group 1 (pulsatile) (n:50)	Group 2 (non-pulsatile) (n:50)	*P*-value
Clock drawing test	Preoperative	5.64 ± 0.49	5.54 ± 0.5	0.312
	Postop. 1^st^ day	5.4 ± 0.54	4.66 ± 0.52	0.001*
	Before discharge	5.66 ± 0.48	5.44 ± 0.5	0.001*
Standardized mini mental test	Preoperative	28.14 ± 1.18	28 ± 1.25	0.569
	Postop. 1^st^ day	27.64 ± 1.05	24.44 ± 1.64	0.001*
	Before discharge	27.92 ± 1.16	24.66 ± 1.22	0.001*

Mann-Whitney U test

* *P*<0.01

Although the intubation times were similar in the postoperative follow-up of the groups, the intensive care stay was recorded as longer in Group 1, and the hospital stay was longer in Group 2. These data were found to be lower in the pulsatile group. No severe and prolonged hypotensive picture occurred in the intraoperative and postoperative period in any of the patients in the two groups. Similar results were found in terms of the frequency of atrial fibrillation in the postoperative follow-ups (*P*=0.218). No difference was found between the groups in terms of major complications and early mortality. There was no difference between the patients in terms of routine laboratory data in the postoperative period (Table 5). There was no significant difference between the biochemical parameters, kidney function test values, in the postoperative period in both groups. However, when the hemogram values were examined, only postoperative day five white blood cells values were found to be significantly lower in the pulsatile group (*P*=0.039).

**Table 5:** Postoperative follow-up and laboratory parameters.

Parameters	Group 1 (pulsatile) (n: 50)	Group 2 (non-pulsatile) (n: 50)	*P*-value
Intubation time (hour)	8.83 ± 6.88	8.52 ± 9.36	0.478
ICU follow-up time (hours)	70 ± 28.17	53.26 ± 18.87	0.001*
Hospitalization time (days)	6.66 ± 1.12	7.62 ± 1.98	0.001*
Postop. 1^st^ day			
Hgb (g/dl)	9.08 ± 0.9	9.14 ± 1.17	0.619
Hct (%)	27.37 ± 2.83	26.92 ± 3.02	0.512
Creatine (mg/dL)	1.47 ± 3.02	1.07 ± 0.28	0.956
BUN (mg/dl)	24.62 ± 6.53	24.67 ± 8.41	0.544
KCl (mEq/L)	4.13 ± 0.24	4.11 ± 0.42	0.718
Na (mmol/L)	141.84 ± 3.25	142.6 ± 4.92	0.516
Ca (mg/dL)	8.63 ± 0.52	8.69 ± 0.61	0.689
Postop. 5^th^ day			
Hgb (g/dl)	9.46 ± 0.72	9.35 ± 0.9	0.286
Hct (%)	29.39 ± 1.96	29.24 ± 2.15	0.761
Creatine (mg/dL)	1.16 ± 0.17	1.14 ± 0.2	0.519
BUN (mg/dl)	26.24 ± 6.34	26.48 ± 6.33	0.992
KCl (mEq/L)	4.06 ± 0.37	4.01 ± 0.27	0.911
Na (mmol/L)	139.98 ± 2.7	139.3 ± 2.12	0.065
Ca (mg/dL)	8.92 ± 0.75	8.63 ± 0.42	0.073
Blood transfusions			
ES (n)	2.34 ± 0.63	2.42 ± 0.61	0.204
FFP (n)	2.39 ± 0.76	2.35 ± 0.54	0.358

BUN=blood urea nitrogen; ES=erythrocyte suspension; FFP=fresh frozen plasma; Hct=hematocrit; Hgb=hemoglobin; ICU=intensive care unit; KCl=potassium chloride

* Mann-Whitney U test

**Fig. 1:**
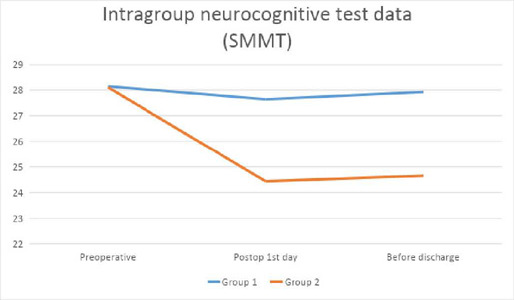
Intragroup neurocognitive test data. SMMT=standardized mini mental test.

While the neurocognitive test results of the groups are compared in Table 4, the comparison within each group is made in Figures 1 2. CDT and SMMT test values were compared within the groups as preoperative, postoperative day 1, and before discharge. In the non-pulsatile group, a significant difference was detected between preoperative values and postoperative day 1, and between preoperative values and predischarge values. These results were compared with parallel curves (Figure 1 2).

**Fig. 2:**
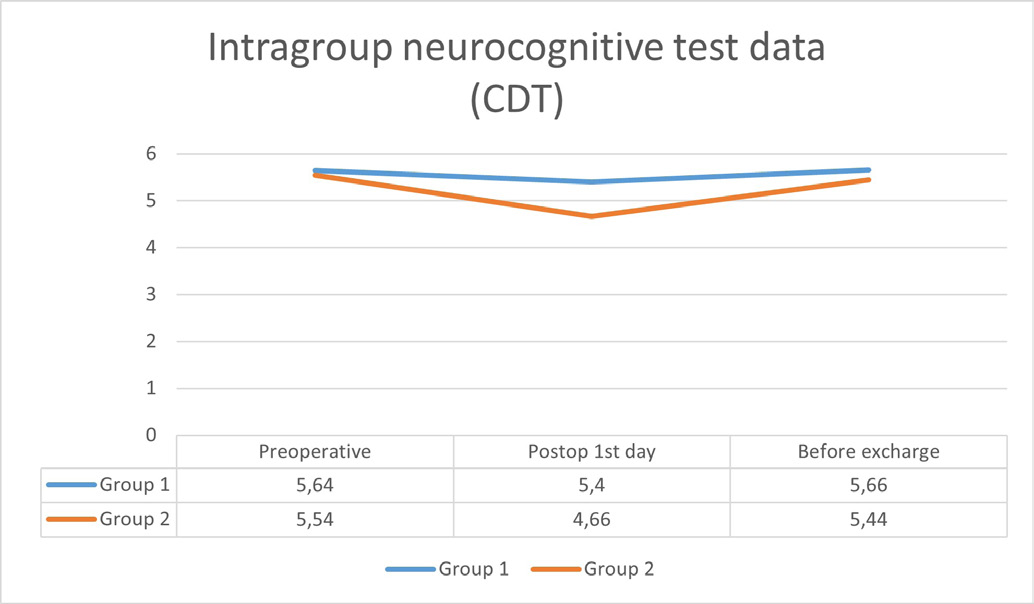
Intragroup neurocognitive test data. CDT=clock drawing test.

## DISCUSSION

Although a safe anastomosis environment is provided for coronary revascularization during open heart surgery, some complications may occur due to the heart-lung machine and ECC frequently used in this period. Despite the development of surgical techniques and the increase in the experience of surgical teams, the frequency of these complications is still at a higher level due to the fact that older patient groups are operated today. Particular attention should be paid to cerebral complications, since it causes serious increases in mortality and morbidity. Although it is not taken as seriously as major cerebrovascular events, the decline in cognitive functions can continue for a long time and cause a decrease in quality of life^[7]^. While it is easier to diagnose severe cerebral involvement with radiological examinations, it is not possible to detect the effect on cognitive functions by radiological methods. Therefore, we thought to make this evaluation with tests developed by neuropsychiatrists (SMMT, CDT). In this study, the main goal is to reveal whether neurocognitive functions are affected in the early period due to flow differences with simple neurocognitive function tests.

It is known that there are serious changes in cerebral blood flow (CBF) during CPB, which is used in open heart surgery^[8]^. It is also known that pulsatile and non-pulsatile currents cause significant differences in organ perfusions^[9,10]^. There are a lot of studies showing that the pulsatile flow is more suitable for physiology. In a study by Ündar et al.,^[11]^ it was shown that there was an increase in CBF, higher cerebral metabolic rate, higher cerebral oxygen delivery, and lower cerebral vascular resistance^[12,13]^. Tovedal et al.^[14]^ reported that they could not see a significant difference in cerebral flow in their study using near infrared spectroscopy (or NIRS).

Major neurological complications that develop after cardiac surgery are not difficult to recognize, but they are difficult to name because there is no diagnostic criterion for neurocognitive dysfunction, which is one of the minor complications^[15]^. We thought that we could evaluate these functions with CDT and SMMT tests accepted by international studies. Performing these tests before and after the operation and evaluating the changes gave us the opportunity to comment on neurocognitive functions through these tests.

The etiology of neurocognitive dysfunction occurring in the postoperative period has not been fully elucidated. However, neuroinflammation is thought to be an important factor^[16]^. Surgical trauma is a trigger for the initiation of the inflammatory response. Irregularity of this response may lead to the formation of neuroinflammatory response and consequently to regression in cognitive functions in the postoperative period^[17]^. A general inflammation process that starts with the contribution of the heart-lung machine causes the deterioration of endothelial functions. The process that starts in this way may lead to increased permeability in the blood-brain barrier and damage to neurons^[18]^. It has been shown in some studies that it will be beneficial in terms of less exposure to this inflammatory process, as it is more suitable for normal human physiology to continue the flow in a pulsatile manner during cardiac arrest in the course of the operation^[10,19]^. The fact that the results of postoperative neurocognitive tests were better in the group with pulsatile flow in our study supports these studies in the literature. Although similar results were obtained in both groups in the tests performed in the preoperative period, the differences between the groups in the tests performed in the postoperative period suggest that operational reasons are effective. The lack of significant differences in the analysis of intraoperative data also supports this view. In the study conducted by Deiner S et al.^[20]^, it is stated that there should be a 25% decrease in SMMT values to evaluate neurocognitive function loss. In addition, it is known that CDT values must be < 4 to be considered a loss of neurocognitive function. Although we did not have any patients with severe neurocognitive dysfunction in our study data, we found that these values were meaningful in comparing the groups. We think that these results will make a significant contribution to us, especially for patient groups that are likely to experience neurocognitive function loss.

Since the decline in cognitive functions also reduces the patient’s compliance with the health care team, it may cause some difficulties in terms of those who should pay attention during postoperative intensive care and service follow-ups. In our study, we think that this was effective in the longer intensive care and service follow-up periods of the patients in the non-pulsatile group.

Eighty percent of neurological deficits that develop after CPB return to normal within a long period of six months to five years^[21]^. In a study by Milne et al.^[22]^, decrease in cerebral oxygen saturation was found to be an important factor in the occurrence of neuropsychological dysfunction. In our study, the lack of difference between oxygen saturation values in intraoperative blood gas follow-ups strengthened our opinion that the type of flow influenced the difference in neurocognitive tests. In the healthy adult brain, under maintained physiological and normal intracranial pressure conditions, CBF is constant over a definite range of perfusion pressure, *i.e.,* systemic blood pressure. This is known as cerebral autoregulation and provides approximately 50 ml of CBF per 100 g of brain tissue^[23]^. It is accepted that this autoregulation system is preserved during mild hypothermia applied during cardiac surgery^[24]^. The fact that there was no difference in intraoperative body temperature between the two groups in our study minimized the effect of this issue in terms of cerebral functions.

Advanced age, duration of cross-clamping and CPB, length of stay in intensive care unit, and length of hospital stay are considered as risk factors for neurocognitive dysfunctions after coronary artery bypass^[25]^. In our study, there was no difference in age between the two groups. This has made an important contribution to the comparison of the groups with the pump flow direction. The postoperative intensive care follow-up period of the patients in the non-pulsatile group was significantly longer. Since no significant difference was observed in terms of hemodynamic parameters during the follow-up periods, we think that the intensive care follow-ups of these patients were prolonged due to compliance problems with the healthcare team.

### Limitations

There are studies showing that neurocognitive functions seen after cardiac surgery largely disappear within six months after the operation. In our study, follow-ups were conducted for short periods of time. There is no data including long-term results.

Neurocognitive dysfunctions were determined by internationally accepted tests. However, no study has been conducted in terms of laboratory values to evaluate neuroinflammation.

## CONCLUSION

After all these data and statistical analyses, we believe that pulsatile flow should be preferred especially in high-risk patient groups in terms of neurological complications. Thus, we think that these patients will have less adjustment problems in the postoperative period and their neurocognitive functions will be better preserved.

## Abbreviations, Acronyms & Symbols

BMI: Body mass index
BSA: Body surface area
BUN: Blood urea nitrogen
CBF: Cerebral blood flow
CDT: Clock drawing test
COPD: Chronic obstructive pulmonary disease
CPB: Cardiopulmonary bypass
CRF: Chronic renal failure
DM: Diabetes mellitus
ECC: Extracorporeal circulation
EF: Ejection fraction
ES: Erythrocyte suspension
EuroSCORE: European System for Cardiac Operative Risk Evaluation
FFP: Fresh frozen plasma
Hct: Hematocrit
Hgb: Hemoglobin
ICU: Intensive care unit
KCl: Potassium chloride
MAP: Mean arterial pressure
PAD: Peripheral artery disease
SMMT: Standardized mini mental test
